# Myeloid-Derived Suppressor Cells in Pregnancy and the Neonatal Period

**DOI:** 10.3389/fimmu.2020.584712

**Published:** 2020-10-09

**Authors:** Natascha Köstlin-Gille, Christian Gille

**Affiliations:** Department of Neonatology, University Children's Hospital Tuebingen, Tübingen, Germany

**Keywords:** myeloid-derived suppressor cells (MDSC), pregnancy, materno-fetal tolerance, neonate, tolerance, microbiome

## Abstract

During pregnancy, the immune systems of mother and offspring are challenged by their close adjacency to balance tolerance and rejection. After birth the neonate has to continue this balance towards its new environment by tolerating commensals while rejecting pathogens and towards its developing tissues to avoid inflammatory damage while overcoming immunosuppression. Our group was the first to link immunosuppressive features of myeloid derived suppressor cells (MDSC) to materno-fetal tolerance, neonatal susceptibility to infection and inflammation control. Here we summarize recent advances in this dynamic field.

## Introduction

Discrimination between self and non-self is one of the fundamental features of the mammalian immune system. Especially during pregnancy, the immune systems of both, the mother and the fetus perfectly balance between protection against pathogens and tolerance towards a semi-allogeneic organism. Dysfunction of the immune adaptation during pregnancy can lead to severe complications like pregnancy loss, preeclampsia, preterm birth or fetal growth restriction. Initially the field of materno-fetal tolerance mainly focused on T cell immunology (1) [reviewed in (2)], however it has become more and more clear that the immune mechanisms leading to successful pregnancy are much more complex and that our understanding of how this exceptional situation is facilitated exhibits significant gaps.

After birth, the neonatal organism is challenged to adapt immunological functions, when body surfaces become colonized with microbes, directly exposing the neonatal immune system to potential pathogens, also requiring a perfect balance between defense against pathogens and tolerance towards commensals.

Myeloid-derived suppressor cells (MDSC) are myeloid cells with the ability to suppress various types of immune responses. While other myeloid cells such as monocytes, macrophages, dendritic cells (DCs), polymorphnuclear (PMN) neutrophils, eosinophils, and basophils classically get activated by strong signals through pathogen-associated molecular patterns (PAMPs) or danger-associated molecular patterns (DAMPs) resulting in a pro-inflammatory response, MDSC rather expand under conditions with chronic infection or inflammation and act anti-inflammatory (3). MDSC mainly consist of two cell types named granulocytic MDSC (GR-MDSC) with phenotypic similarity to neutrophils and monocytic MDSC (MO-MDSC) with phenotypic similarity to monocytes, to date making it impossible to clearly identify MDSC only by phenotypic characteristics. In mice, GR-MDSC are defined as CD11b^+^/Ly6G^+^/Ly6C^lo^ and MO-MDSC as CD11b^+^/Ly6G^-^/Ly6C^hi^ cells. In humans, GR-MDSC expresses the granulocytic markers CD11b, CD15 and/or CD66b, but are negative for the monocytic surface antigen CD14. They can only be distinguished from granulocytes by their sedimentation in the low-density fraction (peripheral blood mononuclear cells, PBMC) after density gradient centrifugation. Recent data suggest, that expression of the lectin-type oxidized low density lipoprotein (LDL) receptor 1 (LOX-1) might be a possible marker to distinguish between granulocytes and GR-MDSC. Human MO-MDSC are defined as CD14^+^/HLA-DR^lo/−^; their discrimination from monocytes is based on their generally lower expression of HLA-DR (4–6). However to date, GR-MDSC and MO-MDSC have to be identified by their suppressive capacity, primarily towards T-cell proliferation.

MDSC use a large number of mechanisms to suppress immune responses. Among these, the best known are a depletion of the essential amino acid arginine by expression of the enzymes Arginase I (ArgI) and inducible nitric oxide synthase (iNOS), the sequestration of cysteine by indoleamine 2,3-dioxygenase (IDO), the production of anti-inflammatory cytokines like TGFβ and IL-10, the synthesis of prostaglandins by expression of cyclooxygenase-2 (COX-2) and the production of reactive oxygen species (ROS) [reviewed in (7)]. The main targets of MDSC-mediated immune suppression are T-cells, however also a suppression of NK-cell functions (8) and DC functions (9), as well as a modulation of monocyte/macrophage functions (10) have been reported. Furthermore, MDSC can induce regulatory T-cells (11), which in turn exert their own type of immune suppression.

Accumulation and activation of MDSC are driven by various factors. Condamine et al. proposed a two-signal model in which the first signal leads to an expansion of myeloid cells and inhibition of their differentiation and the second signal converts these immature cells to MDSC (3, 12). Factors involved in this process are growth factors and cytokines leading to activation of transcription factors such as signal transducer and activator of transcript 1, 3 and 6 (STAT1, 3, and 6), CCAAT/enhancer binding protein β (C/EBPβ) or NOD-, LRR- and pyrin-domain containing protein 3 (NLRP3) [reviewed in (12)].

Primarily, MDSC accumulation has been described under tumor conditions, leading to inhibition of the immune response against tumor cells and to spreading of the disease (13). In the following years, MDSC accumulation has been described under various other conditions such as sepsis/infection, trauma, autoimmune diseases, obesity, ageing and transplantation where they seem to play either a detrimental or a beneficial role [reviewed in (14)]. Overall, it appears that under conditions, where immune tolerance is needed to survive, MDSC accumulation may be advantageous for the host. In this review, we aim to summarize data on MDSC during perinatal time, i.e. during pregnancy and the neonatal period, as a phase of life, where under physiological conditions tolerance is needed most.

## MDSC During Pregnancy

### MDSC During Normal Pregnancy

The first description of a MDSC-accumulation during pregnancy came from Mauti et al. who showed that increased permissiveness for tumor metastasis during gestation in mice was accompanied by an expansion of MDSC with inhibitory effects on NK-cell activity and that depletion of MDSC in pregnant mice reduced metastasis (8). Later, our group showed, that also during physiologic conditions in healthy human pregnancies an accumulation of GR-MDSC but not MO-MDSC occurred with up to tenfold higher numbers of GR-MDSC in the peripheral blood of pregnant women compared to blood of healthy non-pregnant controls (15). Levels of GR-MDSC were highest during early gestation (15, 16) and dropped within a few days postpartum to levels of non-pregnant women (15). In human placenta GR-MDSC were shown to be enriched in comparison to maternal and fetal blood (17) and mainly located in decidua and intervillous space (17, 18). Genetic analyses revealed that they descend from maternal origin (17). Pan et al. and Zhang et al., showed that also MO-MDSC increased in the peripheral blood of pregnant women (19, 20), however percentages of MO-MDSC were much lower than that of GR-MDSC.

In accordance with the data from humans, it has been shown that GR-MDSC also expand during murine pregnancy both in the periphery (21, 22) and in the uterus (22–24), especially during early gestation (21, 24). This phenomenon was observed in syngeneic (8, 22, 23) as well as in allogeneic (21, 24, 25) murine pregnancies. Mouse strains used were BALB/c (21), C57BL/6J (8, 22, 25), CBA/J (24, 25), and FBVn (23).

### MDSC During Pathological Pregnancies

It was shown that women with miscarriage had decreased levels of GR-MDSC both in blood (16) and in placenta (16, 17, 26), while numbers of MO-MDSC did not differ (26). Studies in mice revealed that MDSC accumulation during pregnancy was also decreased in abortion prone animals (24, 25, 27) and adoptive transfer prevented fetal rejection in the murine abortion model (25). In addition, depletion of MDSC caused gestation failure (21, 23–25). Interestingly, Ostrand-Rosenberg et al. showed that especially MDSC-depletion at day 4.5 (E4.5) of murine pregnancy, which is the time of implantation, completely prevented successful pregnancy, while MDSC-depletion after E8.5 did not affect pregnancy rates (21). This is in line with two human studies showing that high GR-MDSC levels predict a better outcome after in-vitro fertilization (28, 29). One study investigated MDSC in preeclampsia and showed that GR-MDSC, but not MO-MDSC levels are decreased in peripheral blood and cord blood of preeclampsia patients in comparison to healthy pregnancies (30), however functional studies are lacking.

### MDSC Functions and Suppressive Mechanisms During Pregnancy

**Figure 1:**
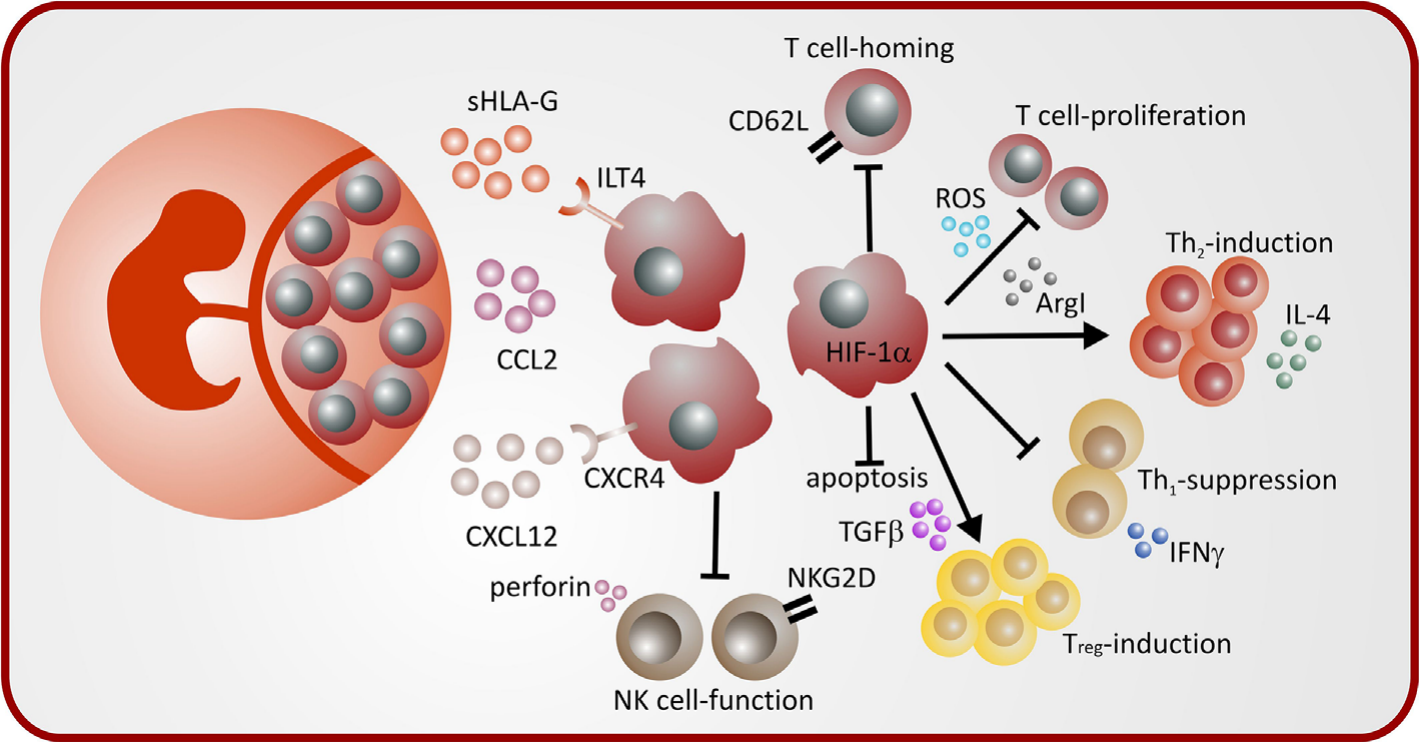
Mechanisms of MDSC induction and MDSC-mediated immune modulation during pregnancy.

Pregnancy induced MDSC from different compartments (peripheral blood, uterus, decidua) exert different effector mechanisms to modulate immune effector cells during pregnancy. A summary is depicted in **Table 1** and **Figure 1**.

**Table 1:** Origin, effects and mechanisms of MDSC in pregnancy.

MDSC origin	Effect	Main mechanism of MDSC	Ref.
GR-MDSC from peripheral blood	Inhibition of T-cell proliferation	–	(15, 17, 18, 31)
	Downregulation of CD3 ζ chain	ArgI	(31)
	Inhibition of T-cell proliferation	ROS	(25)
GR-MDSC from placenta (human)	Inhibition of Th1 response	Cell contact	(17)
	Induction of Th2 response	Cell contact	(17)
	Inhibition of T-cell proliferation	ROS	(25)
MDSC (murine)	Inhibition of T-cell proliferation	ROS	(25)
	Decrease of uterine T-cells	–	(21, 23)
	Decrease of T-cell activation, L-selectin downregulation	–	(21)
	Induction of Tregs	TGF-beta Beta-catenin	(24)
	Reduce NK-cell cytotoxicity, inhibition of perforin, downregulation of NKG2D	–	(8, 32)

Source of MDSC, effect on immune effector cell and the main MDSC mechanisms are listed. ArgI, arginase I; ROS, reactive oxygen species; iNOS, inducible NO-synthetase.

#### Inhibition of T-Cell Function

Suppression of T-cell function is a main feature of MDSC. Changes in T-cell function are one of the best examined mechanisms mediating maternal-fetal tolerance (33). In humans, it could have been shown that GR-MDSC during pregnancy inhibit T-cell proliferation (15, 17, 18), express ArgI, iNOS and IDO and produce ROS (15, 18, 30). ArgI expression led to downregulation of CD3ζ chain on T cells and to decreased T-cell proliferation (31), while inhibition of ArgI restored T-cell proliferation *in-vitro* (17). Furthermore, it was shown that GR-MDSC isolated from placenta exhibited marked increased ROS-production in comparison to GR-MDSC from the periphery (17). In pregnant mice, the main mechanism of T-cell suppression by GR-MDSC was ROS-production (25). From tumor-bearing mice, it is known that induction of antigen-specific T-cell tolerance is mediated by MDSC via ROS (34), indicating that increased ROS-production could be also a mechanism for suppression of specific T-cell-immunity against fetal antigens.

In mice, Zhao et al. showed that depletion of MDSC during pregnancy led to an increase in uterine T-cells (23). Later, Ostrand-Rosenberg et al. showed, that the detrimental effect of MDSC-depletion during pregnancy was mediated by an increase in T-cell activation and an upregulation of L-Selectin supposing that MDSC prevent homing of alloreactive T-cells to the uterus (21).

#### Modulation of T-Helper Cell Function

Besides a general suppression of T-cell responses, MDSC during pregnancy also modulate polarization of Th-cells. Different studies described a predominance of Th2 responses and a suppression of Th1-responses [reviewed in (35)] as well as an accumulation of Tregs during normal pregnancy (36). Our group showed *in-vitro* that placental GR-MDSC induce Th2-responses and inhibit Th1-responses in a cell-contact dependent manner (17). Furthermore, Kang et al. showed that GR-MDSC from pregnant mice induced Tregs via production of TGF-β and the transcription regulator β-catenin (24). In contrast to that, Ren et al. described an increase in Tregs after MDSC-depletion during pregnancy.

#### Inhibition of NK Cell Function

NK cells are the predominant cell type in deciduae of early pregnancies [reviewed in (37, 38)] and MDSC have been shown to suppress NK-cell functions under tumor-conditions (39, 40). During pregnancy, MDSC may contribute to reduced NK-cell cytotoxicity systemically (8), as well as locally in the uterus (32). Mechanisms for inhibition of NK cell function by MDSC during pregnancy are an inhibition of perforin-cytotoxicity and a downregulation of the surface receptor NKG2D on NK-cells (32).

#### Effects on Myeloid Cells

Decidual DCs have been shown to be in a tolerogenic state with reduced co-stimulatory capacity and altered cytokine expression [reviewed in (41)]. Hu et al. showed that in hepatocellular carcinoma MDSC impair DC function (42). To our knowledge, until now no data exists concerning the influence of MDSC on myeloid cell function during pregnancy. Zhao et al. described an increase in infiltrating DCs in the uteri of pregnant animals after MDSC-depletion, while total myeloid cell numbers remained unchanged (23), however functional data is missing.

### MDSC Expansion and Activation During Pregnancy

#### Soluble Factors Regulating MDSC Expansion and Activation During Pregnancy

Different studies investigated potential mechanisms regulating MDSC accumulation and activation during pregnancy. Zhang et al. showed *in vitro* and in human cells an expansion of and an upregulation of Arg1 in MO-MDSC by the human trophoblast cell line HTR8 via CCL2 (20). Pan et al. showed an induction of MO-MDSC by 17β-Estradiol also in human cells (19). In murine cells, progesterone but not estrogen induced MDSC *in-vitro*. Our group showed that co-culture with the human trophoblast cell line JEG-3 induced MDSC. This was partially mediated through the receptor CXCR4 on MDSC, indicating that CXCL12, the ligand of CXCR4, plays a role in MDSC-expansion (17). Furthermore, we showed that soluble human leucocyte antigen G (sHLA-G) induced MDSC from PBMC through the receptor immunoglobulin like transcript (ILT) 4, increased suppressive activity of GR-MDSC and induced IDO expression (43).

*In-vivo*, Ostrand-Rosenberg et al. demonstrated that the IDO-inhibitor 1-methyltryptophan (1-MT) decreased the level of MDSC in pregnant mice, while application of granulocytic colony-stimulating factor (G-CSF) restored MDSC-levels (21). Studies describing soluble factors mediating MDSC induction or activation *in-vivo* that may be targets for therapeutic interventions are still lacking.

#### Transcriptional Regulation of MDSC Expansion and Activation During Pregnancy

Three studies described a role of STAT3 for regulation of MDSC function during pregnancy. STAT3 has been shown to be involved in MDSC expansion via estradiol and via progesterone (19, 25) as well as in HLA-G-mediated MDSC-activation (43). Furthermore, our group showed that lack of the transcription factor hypoxia-inducible factor 1α (HIF-1α) led to decreased accumulation of MDSC during pregnancy and impaired suppressive activity (22). Interestingly, mice with homozygous deletion of heme oxygenase 1 (HO-1), one of the target genes of HIF-1α (44) are infertile and heterozygous deletion results in preeclampsia like pregnancies with primarily absorption of HO-1–deficient fetuses (45, 46). Furthermore, it has been shown that HO-1 is relevant for MDSC function during transplantation (47). The role of HO-1 for MDSC function during pregnancy remains to be elucidated. A summary of mechanisms leading to MDSC accumulation and activation during pregnancy is depicted in **Figure 1**.

## MDSC in the Fetus and Neonate

### MDSC in Cord Blood

Concordant to the maternal side, MDSC have also been found in cord blood, suggesting that they not only modulate maternal but also fetal immune system to ensure feto-maternal tolerance (48). In two cohorts comprising cord blood samples from 83 healthy, full-term neonates after caesarian section, MDSC accounted for about 5% (0,3% - 60%) of mononuclear cells (48, 49) - levels formerly only described in pathological processes. Accumulated cord blood MDSC were characterized as positive for granulocytic markers CD66b or CD15, positive for CD33, CD11b and IL-4Ra and negative for CD14 and HLA-DR, thus classified as GR-MDSC, while MO-MDSC were not elevated.

Several clinical characteristics have been evaluated as influencing factor for cord blood GR-MDSC accumulation. Most interesting, cord blood GR-MDSC levels seem to be independent of gestational age (50), resembling GR-MDSC rates in maternal blood. However, it has been reported, that very low birth weight infants, i.e. infants with a birth weight below 1500 g and small for gestational age (SGA) infants, i.e. weight below the 10^th^ percentile for the gestational age may have decreased cord blood GR-MDSC levels (50, 51). No influence has been described for biological sex and exposition to prenatal medication of the mother such as magnesium or corticosteroids, influencing factors that have been described for other regulatory immune effector cells such as regulatory T cells (Treg) in preterm infants (52).

While until now, no data on MDSC counts are available directly from the fetal blood, cord blood MDSC are regarded as of fetal origin. This is supported by the fact, that GR-MDSC have been shown to be elevated in cord blood not only from term, but also from preterm infants as early as 23 weeks of gestation (50).

Elevation of MDSC has also been described in neonatal mice in spleens and bone marrow (51), with substantial expansion of GR-MDSC up to 40% while MO-MDSC were only marginally elevated.

### MDSC After Birth

After birth, different studies showed elevated GR-MDSC levels persist for at least 4 to 6 weeks, accounting a fraction of 2% to 4% of PBMC with a negative correlation of MDSC-counts and postnatal age. MDSC then further decreases to adult levels during the second month of life (49, 50). Paralleled to the kinetics in humans, in neonatal mice, GR-MDSC levels stay elevated during the first 3 weeks of life, then dropping to adult levels (51).

The parallelism in the kinetic of postnatal persistence of neonatal MDSC in human and mice is somehow astonishing since adaptation and maturation of various immunological functions is generally thought to follow different time scales in men and mice and are different in each immunological compartment such as innate cytokine response, innate myeloid cell populations and adaptive immune functions (53). For neonatal MDSC persistence external factors regulating MDSC accumulation under other circumstances such as tumor environment may be relevant. Intriguingly, reports on CD71^+^ erythroid cells, another immature cell population with immunosuppressive activity, which are highly elevated in cord blood and peripheral blood during the first weeks of life and thus extraordinarily mimicking the course of MDSC accumulation (54) hint toward a role of these cells for microbiome establishment after birth. Taken together, neonatal MDSC might not only be interpreted as remnant of materno-fetal tolerance and as a sign of distinct immune adaptation, but might be a crucial regulator of inflammation during the neonatal period.

### MDSC in Neonatal Pathologies

Data from our group showed, that in neonates suffering from bacterial infection GR-MDSC may dramatically expand, even from the elevated level seen in healthy neonates (50), and that GR-MDSC levels correlated with inflammatory markers such as C-reactive protein (CRP), demonstrating the influence of an inflammatory environment of MDSC at least on their expansion, as seen in adult patients [reviewed in: (55)].

Interesting but somehow unclear data descend from an inflammatory disease model of neonatal mice using a gavage/hypoxia approach to induce necrotizing enterocolitis (NEC), an often-devastating inflammatory bowel-disease of preterm infants. Selective depletion of MDSC with an agonistic antibody to Tumor-Necrosis-Factor-Related Apoptosis-Inducing Ligand receptor (TRAIL-R) depleted MDSC from the lamina propria and led to shortened survival and pronounced intestinal inflammation and bacterial load (51) during murine NEC. In contrast, neonatal splenic myeloid cells containing MDSC adoptively transferred intraperitoneally greatly decreased these symptoms compared to adult mouse spleen cells or mock transfer (51). These data may underline the potential role of neonatal MDSC as regulator of inflammation during the neonatal period.

### MDSC Functions in Neonates

Functional characteristics of cord blood MDSC and neonatal MDSC have been described in some details and have added several new aspects in the knowledge of MDSC functionality in general (**Table 2**). Besides the ability to inhibit T cell proliferation as prerequisite and characteristic function of MDSC (48), we could demonstrate that cord blood GR-MDSC preferentially inhibited Th1 cells, contrarily polarized towards Th2 reactions (56) and induced Tregs (51, 56). Intriguingly these processes seemed to be mediated by different effector mechanisms. Th1 inhibition required direct cell-contact and was independent of other cell types, while induction of Th2 cells was mainly mediated through soluble factors, i.e. ArgI and ROS. Induction of Tregs was partially mediated through iNOS expression (56).

**Table 2:** Origin, effects, and mechanisms of MDSC in neonates.

MDSC origin	Effect	Main mechanism of MDSC	Ref.
GR-MDSC from cord blood	Inhibition of T-cell proliferation	–	(44, 45)
	Inhibition of Th1 responses	Cell contact	(51)
	Induction of Th2 responses	ArgI, ROS	(51)
	Induction of Tregs	iNOS	(47, 51)
	Downregulation of HLA-molecules on monocytes	–	(52)
	Upregulation of co-stimulatory molecules PD-L1, PD-L2	–	(52)
	Decrease of TNF-alpha, IL-1beta	–	(52)
	Induction of IL-8	–	(52)
	Inhibition of NK-cell cytotoxicity	–	(44)

Source of MDSC, effect on immune effector cell and the main MDSC mechanisms are listed. ArgI, arginase I; ROS, reactive oxygen species; iNOS, inducible NO-synthetase.

Cord blood GR-MDSC may have effects on other types of immune effector cells such as monocytes and NK-cells. In co-cultures with cord blood GR-MDSC monocytes downregulated HLA class I and class II expression and upregulated co-inhibitory molecules such as programmed death ligand 1 (PD-L1) and PD-L2, showed reduced stimulatory capacity of antigen-dependent and antigen-independent T-cell proliferation and altered cytokine expression upon bacterial stimulation with decreased TNF-α and IL-1β but enhanced IL-8 production (57). Taken together, monocytes seem to be biased towards an immature phenotype typically seen in cord blood monocytes (58). Furthermore, NK cell cytotoxicity was inhibited by cord blood GR-MDSC (48). On the whole cord blood GR-MDSC may orchestrate several other aspects of immune reactions in a way which is thought to be characteristic for the neonatal period.

Concerning other mechanisms for immune suppression/modulation mediated by cord blood GR-MDSC, it could have been shown that cord blood GR-MDSC produce higher amounts of prostaglandin E2 (PGE2),express increased levels of S100A9 and lactoferrin and exert pronounced antibacterial activity compared to adult neutrophils (51).

**Figure 2:**
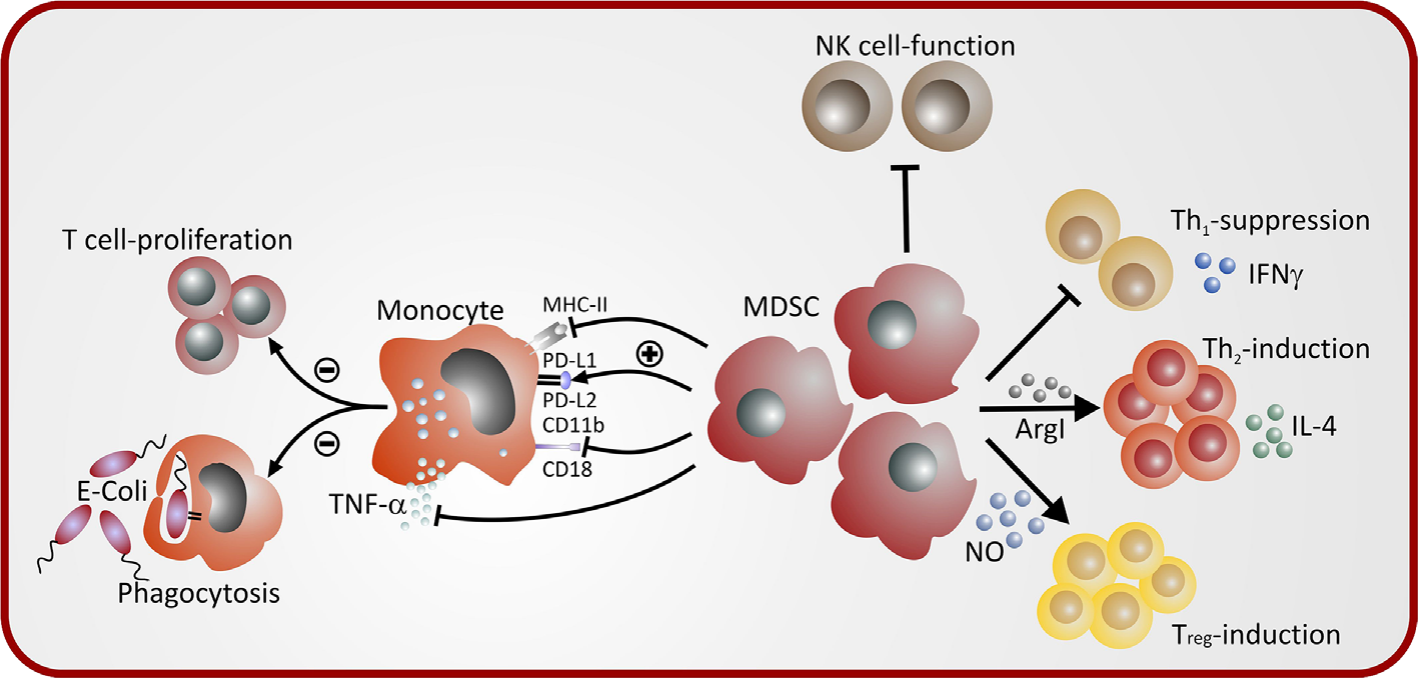
Mechanisms of MDSC-mediated immune modulation in neonates.

While the functionality of neonatal MDSC has not been tested in detail in humans, mainly because of the unavailability of larger volumes of peripheral blood of neonates, function of neonatal murine GR-MDSC and MO-MDSC have been studied in-vitro and in-vivo (51). In-vitro, neonatal GR-MDSC and MO-MDSC exhibited potent suppression of antigen-specific proliferation of OT-1 CD8+ T-cells with stimulated with SIINFEKL antigen as well as antigen nonspecific CD8 T-cell proliferation stimulated with CD3/CD28. *In-vivo*, neonatal GR-MDSC were able to decrease lung inflammation in an ovalbumin-sensitization model (51). A summary of MDSC-mediated immune modulation in neonates is depicted in **Figure 2**.

## MDSC in Breast Milk

Besides the accumulation of MDSC in the maternal organism during pregnancy as well as in the fetal and neonatal organism, recently our group could show that also breast milk contains large numbers of GR-MDSC (59). Numbers of GR-MDSC in breast milk from preterm infants correlated with gestational age (unpublished data). In comparison to GR-MDSC from the periphery, breast milk GR-MDSC were functionally activated with increased T-cell inhibitory capacity, increased expression of CXCR4, PD-L1, PD-L2, and iNOS. Furthermore, we could show that breast milk GR-MDSC suppressed the expression of TLR-4 on monocytes. As putative mechanisms for MDSC-accumulation in breast milk, we investigated if prolactin or oxytocin could induce MDSC from PBMC, but could not show any effect (59). Beyond that, He et al. showed that breastfed infants had increased GR-MDSC levels in comparison to infants fed with formula (51). Further *in-vivo* studies are needed to investigate the functional role of GR-MDSC in breast milk.

## Conclusion

Besides their well described role during pathological processes such as cancer and inflammation, where an expansion of MDSC is detrimental for the host, during the last years MDSC have been discovered also as potent regulators of critical immunological processes during pregnancy and the neonatal period such as maintenance of materno-fetal tolerance and control of inflammation in neonates. Better understanding of the differences between protective and destructive MDSC-populations and the mechanisms leading to their expansion is needed to specifically influence their functions. Targeting MDSC-functions may help to positively impact immunological pregnancy complications as well as inflammatory diseases of the newborn making them an interesting target for cellular based therapies in this field.

## Author Contributions

NK-G and CG both contributed to the scope of the review, literature search, and writing the manuscript. All authors contributed to the article and approved the submitted version.

## Conflict of Interest

The authors declare that the research was conducted in the absence of any commercial or financial relationships that could be construed as a potential conflict of interest.
